# The Effects of Stresses and Interfaces on Texture Transformation in Silver Thin Films

**DOI:** 10.3390/nano12030329

**Published:** 2022-01-20

**Authors:** Nhat Minh Dang, Zhao-Ying Wang, Chi-Hang Lin, Ming-Tzer Lin

**Affiliations:** 1 Graduate Institute of Precision Engineering, National Chung Hsing University Taichung, Taichung 402, Taiwan; d108067005@mail.nchu.edu.tw (N.M.D.); g2001ya@yahoo.com.tw (Z.-Y.W.); daniel90421@hotmail.com (C.-H.L.); 2 Aeronautical Systems Research Division, National Chung-Shan Institute of Science and Technology, Taoyuan 325, Taiwan; 3 iCenter for Advanced Science Technology, National Chung Hsing University, Taichung 402, Taiwan

**Keywords:** texture transformation of thin film, interface energy, strain energy, stress on thin film

## Abstract

Thin metal films are critical elements in nano- and micro-fabricated technologies. The texture orientation of thin films has a significant effect on applied devices. For Face-Centered Cubic (FCC) metal thin films, when the critical thickness is reached, the texture orientation can transform from (111) to (100) based on the model related to the balance between interfacial energy and strain energy. This research focused on the texture transformation of thin films under two conditions: (1) with or without an adhesion layer in the thin film and (2) with or without initial stress applied through a four-point bending load. In the experiment, two samples (silicon/silver and silicon/titanium/silver) were used to apply different initial stress/strain values and different annealing times. After annealing, an X-ray Diffractometer (XRD) was used to ascertain the preferred orientation of the thin films and the percentage of (111) and (100). Finally, Electron Back-Scattered Diffraction (EBSD) was used to observe the grain size of the thin films. The results showed that, regardless of the existence of an adhesion layer, texture transformation occurred, and this was relatively significant with Ti adhesion layers. Further, the initial stress was found to be small compared to the internal stress; thus, the initial stress imposed in the tests in this research was not significantly influenced by the texture transformation.

## 1. Introduction

Thin metal thin films are widely used in microelectronic components, optical components, and mechanical components. Copper and aluminum are often used to connect microelectronic and mechanical components [1,2,3,4,5], while silver is used in optical reflection components [6,7,8]. Gold is often used to produce the capacitance switch of Radio frequency Microelectromechanical Systems (RF MEMS) [9] and is utilized as the surface substrates of self-assembled molecular films [10], while platinum is commonly used for ferroelectric electrodes and high dielectric components [11].

When a thin film is deposited in a different working environment, the working pressure, deposition rate, and substrate temperature will influence the properties of the thin film [12]. In particular, the mechanical properties of a thin film will be significantly influenced by the preferred texture orientation. Previous studies found that FCC metal thin films had texture transformation from (111) to (100) after annealing [13,14,15,16,17,18,19,20,21,22,23,24,25,26]. Due to the crystalgraphic differences, the (111) or (100) texture determines the stress level and the proclivity to distortion, fracture, and delamination.

As the texture transform happens, the properties of thin Ag films will be different, and the optical reflection components will fail. Therefore, the texture transform is essential to control the performance and reliability.

Previous studies of the Carl, Thompson, and Frost (CTF) model [27,28,29], the transformation from (111) to (100) is based on the competition between the interfacial energy and strain energy. The orientation of (111) has the lowest interfacial energy, while (100) has the smallest biaxial modulus and the smallest strain energy. When a fixed elastic strain is given, changes in the thickness of the thin film indicate a reduction of the interfacial energy for each unit volume, with the strain energy per unit volume and the thickness of the film being constant.

Consequently, there exists a critical thickness; if the film is thicker than the critical thickness, regardless of the influence from grain size, the initially preferred orientation of the thin film (111) will be transformed to (100) completely. However, the CTF model is generating questions now. Studies by Sonnweber-Ribic, P. et al. and Arzt et al. [22,29,30,31] discovered that, for film deposits on different substrates, the transformation mechanism was distinctive from the CTF model. In addition, a study by Ellis, E. A. et al. [8] found that the influence of stress is also distinctive from the driving force description in the CTF model. Therefore, further discussion and clarification are needed to understand the driving force model for texture transformation.

This research focused on the texture transformation of thin Ag films. The experiment investigated the influences of interface and different initial stresses/strain imposed and different annealing times on the films of three different thicknesses: 0.5 µm, 1µm, 1.5 µm. The relationships between the texture intensity with different thicknesses, the initial stress/strain, and annealing time were discussed. The experiment added an interface effect using the existence of an adhesion layer of a thin titanium film to study the influences of its interface on the texture structure of the silver film.

## 2. Fabrication and Experiment Methods

### 2.1. Design and Fabrication of the Test Specimens for the Experiment

For the experiment, we chose (100) p 4-inch single-side polished 400 μm silicon wafers. First, a wafer was cut for the test specimens into a 27 × 5 mm shape. Then, acetone, isopropyl-ketone, buffered oxide etch (BOE) etchants, and deionized (DI) water were used to wash the specimens, which were dried after. It was previously shown [29] that the purity of the film plays an important role in this process. To enhance the purity of the test specimens, the experiment utilized electron beam evaporation (EBE) to deposit the thin films.

In a comparatively high vacuum working environment (3–5 × 10^−7^ Torr), the deposited and focused electron beam produced through EBE can heat a targeted place on the element source without heating the whole element. In this way, pollution is avoided, and the plating speed is enhanced. A separate sputter-deposition of silver films was used for comparison. The environment pressure was under 5 × 10^−6^ Torr, and the working pressure was between 5 × 10^−3^ and 5 × 10^−4^ Torr.

**Figure 1:**
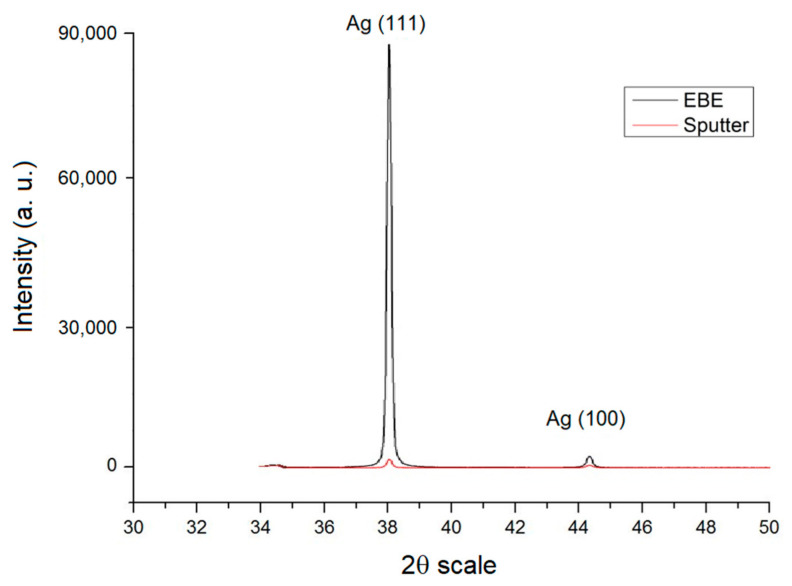
X-Ray diffraction diagram of distinctive depositing methods of thin Ag film 1.5 µm.

We employed an X-ray detection measurement for a geometrical relationship of θ to 2θ to observe the crystal structures of thin films produced by the two depositing methods. The scanning range of the X-ray was 35° ≤ 2θ ≤ 80°. Figure 1 shows the intensity of the crystallographic direction of the 1.5 μm thin Ag film produced by distinctive depositing methods. The thin film was plated by EBE. When 2θ was approximately at 38.14, the intensity of (111) produced was significantly distinctive from that of (100) produced when 2θ was at 44.28. In addition, the intensity of (111) was far larger than that of the thin film deposited by sputtering. The thin film deposited by sputtering had no significant texture intensity compared to that deposited with EBE. Therefore, EBE was used to deposit silver films throughout the experiments.

**Figure 2:**
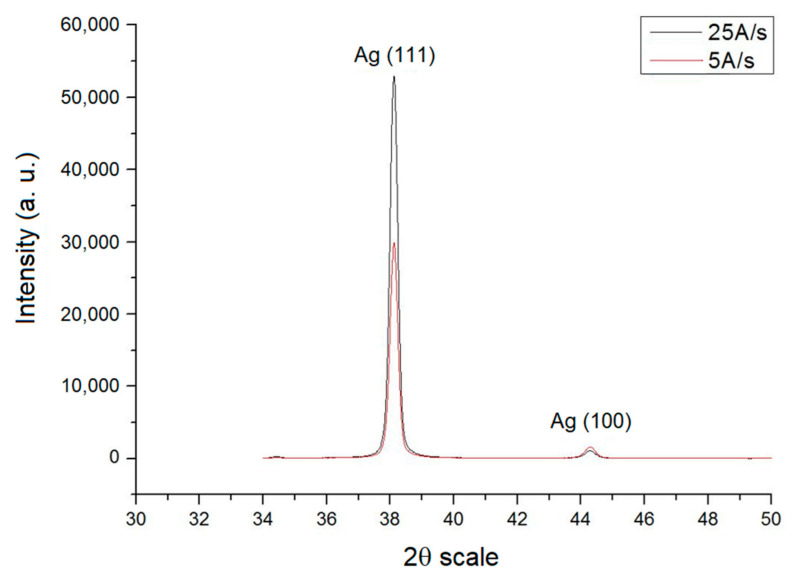
X-Ray diffraction diagram at different plating speeds.

Previous literature [32] mentioned that, at a low deposition rate, a thin Ti film inhibits the movement of atoms to a large area by means of a crystal. When the deposition rate is high, atoms that are absorbed before they reach the lowest surface energy would be blocked by the deposited material. Therefore, when the deposition speed decreases, atoms cannot possibly change due to the atomic diffusion caused by the increase in atomic flow. As demonstrated in Figure 2, when the deposition rate is comparatively low, the intensity of (111) is small. For the convenience of observation, the measured films used to discuss the texture structure transformation studies were all measured at the plating speed of 25 Å per second.

**Figure 3:**
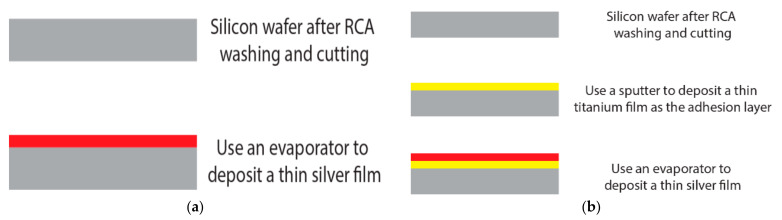
Fabrication of the test specimens (**a**) single thin film (**b**) bilayer thin film with adhesion layer.

From the conclusions drawn above and a previous study [24], the test specimens with a thin Ag film on top of different thicknesses 0.5 µm, 1.0 m, 1.5 µm were fabricated for investigation. Different thicknesses of thin Ag films were made based on mass $d{\bar{M}}_{s}$ falls on the substrate of area $d{\bar{A}}_{s}$ [33] by EBE. Moreover, to compare whether the existence of an adhesion layer influences the texture transformation of the thin silver films, half of the specimens were covered with a 35 nm titanium film as the adhesion layer by sputtering. The depositing parameters are shown in Table 1 and Table 2. The fabrication of the test specimens is shown in Figure 3a,b.

### 2.2. Experimental Method

**Figure 4:**
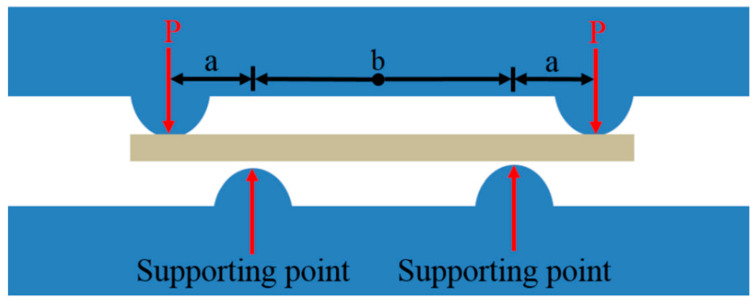
Diagram of a Four-Point Bend moment calculation.

Next, a four-point bend moment platform was used to impose initial stress. As shown in Figure 4, the plane stress on the test specimens can be calculated by

(1) $$\sigma_{f}=\frac{E_{f}}{E_{s}}\sigma_{s}=\frac{E_{f}}{E_{s}}\frac{3mga}{bh^{2}}$$

where

$\sigma_{f}$ is the film stress,

$\sigma_{s}$ is the substrate stress,

$E_{f}$ is the module of the film, and

$E_{s}$ is the module of the substrate.

Based on the above equation and the parameters given in Table 3, the initial stresses imposed in the experiment were 25 and 50 MPa.

After, the annealing experiments were taken inside the YSC DK-45 hot oven (YOTEC INSTRUMENTS Co., Hsinchu, Taiwan) with a temperature of 260 °C. After annealing, a high-resolution X-ray diffractometer Bruker D8 SSS (Bruker Taiwan Co., Hsinchu, Taiwan) was used to measure the texture structure of the thin silver films. The method of θ-2θ was adopted to conduct the measurement. The target material was used to penetrate the X-ray produced by the rotating anode copper target into the test specimens. 

The X-ray then entered the detector using the X-ray diffractometer Bruker D8 SSS (Bruker Taiwan Co., Hsinchu, Taiwan). Based on the wavelength intensity of the crystallographic direction, the measurement results obtained by X-ray diffraction were calculated using proportion. Since other crystallographic directions were not significant, they were neglected, and (111) and (100) were employed as the main calculation objects. The texture fraction calculation is as follows:

(2) $$f_{111}=\frac{I_{111}}{I_{111}+I_{100}}$$

(3) $$f_{100}=\frac{I_{100}}{I_{111}+I_{100}}$$

where

$I_{111}$ is the peak intensity of (111) measured by X-ray diffraction.

$I_{100}$ is the peak intensity of (100) measured by X-ray diffraction.

## 3. Results and Discussion

### 3.1. Texture Changes after Deposition at Room Temperature

**Figure 5:**
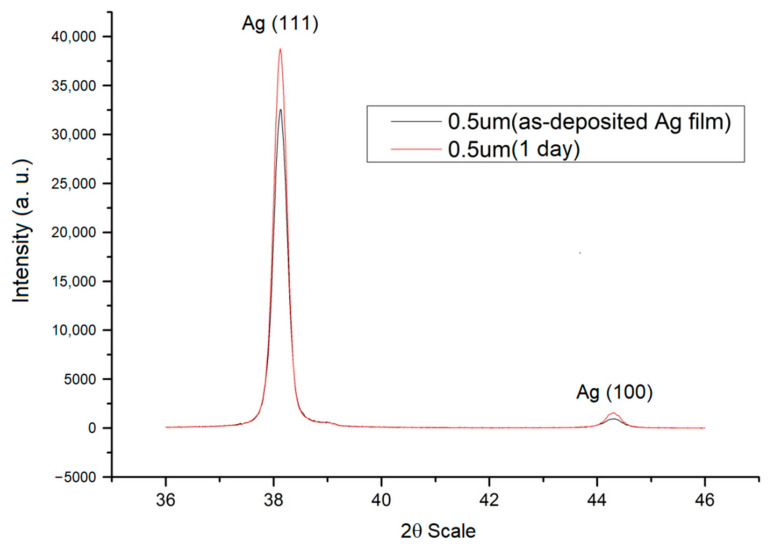
X-ray diffraction diagram of 0.5 μm thin Ag film after plating and being placed at room temperature for one day.

**Figure 6:**
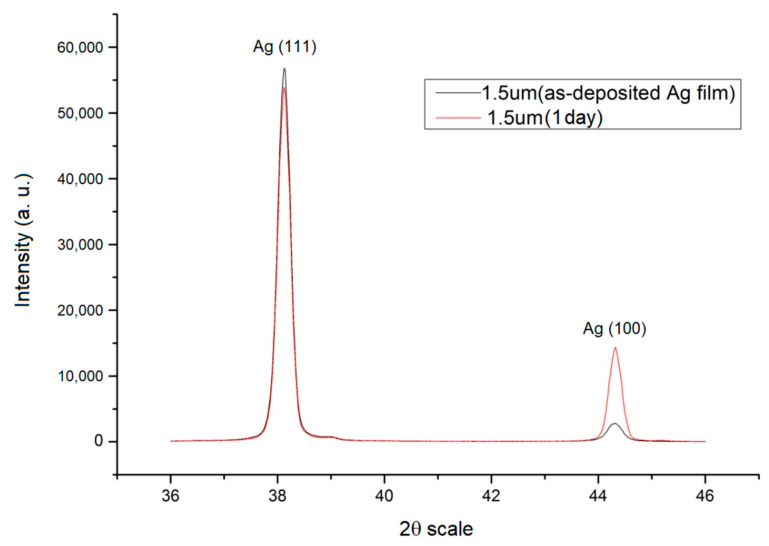
X-ray diffraction diagram of 1.5 μm thin Ag film after plating and being placed at room temperature for one day.

The experimental results showed that the texture structure of the thin Ag film changes at room temperature. Although the preferred orientation was majorly located at (111), we found that if the thin film was comparatively thin, the initial (111) texture would increase. Moreover, when the test specimens were left at room temperature after deposition for hours and days, the textures of the thin films were transformed. In addition, the intensity of the texture transformation from (111) to (100) increased significantly with the thickness increases as shown in Figure 5 and Figure 6.

These results indicated that the texture of the thin Ag films was transformed at room temperature after deposition. Therefore, all test specimens after deposition should be bestowed inside the fridge at a temperature lower than room temperature before the test to avoid texture transformations leading to erroneous conclusions.

**Figure 7:**
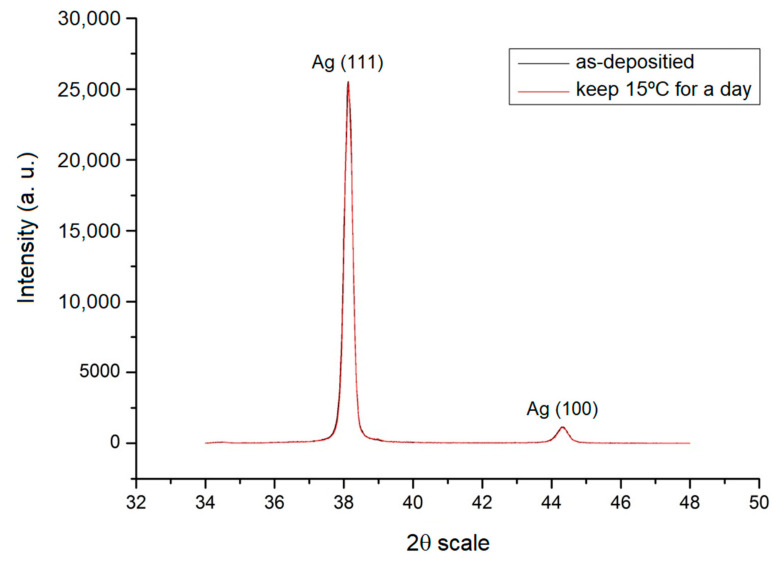
X-ray diffraction diagram of 1.5 μm thin Ag film after plating and being placed in a fridge for one day.

After the deposition, the separated test specimens were immediately put inside a fridge with a temperature of 15 °C. After one day, the test specimens were measured. It can be seen in Figure 7 that the intensity of (111) and (100) showed almost no change. The results in Figure 6, under the same thickness of 1.5 μm, showed that there was a significant and distinctive change. Therefore, all the samples used in this study’s experiments were placed in the refrigerator after deposition.

### 3.2. Influences of an Adhesion Layer

The experiment used the existence of an adhesion layer to explore the influences of adding an interface during the transformation of texture after annealing. A previous study [24] indicated that the texture transformation of a thin Ag film exhibited the same behavior at the annealed temperature of 260, 500 and 600 °C. Therefore, the annealing temperature was set at 260 degrees accordingly [24]. The experimental results found that, for thin Ag films of 1 and 1.5 μm, the texture structure will change regardless of the existence or non-existence of the adhesion layer.

**Figure 8:**
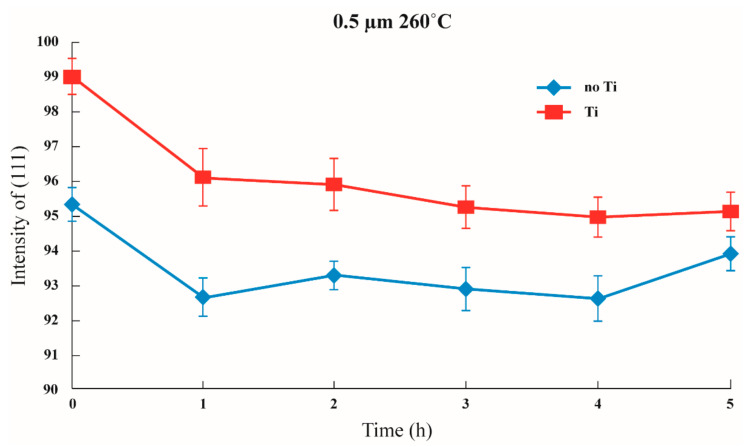
Changes in (111) intensity under the same annealing conditions depending on whether or not an adhesion layer exists in a 0.5 μm thin film.

**Figure 9:**
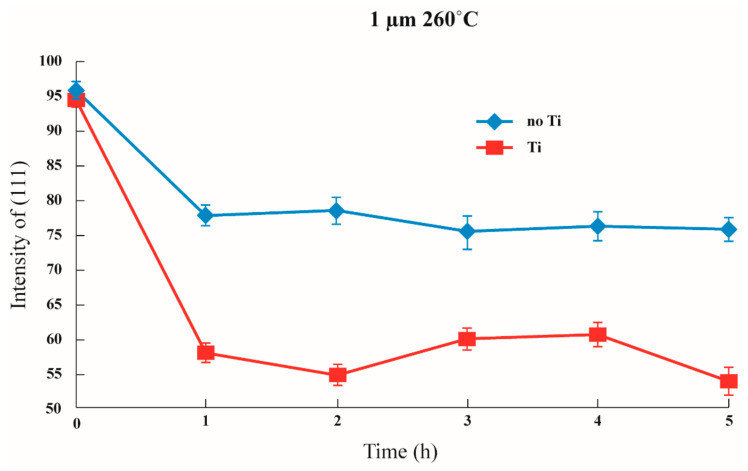
Changes in (111) intensity under the same annealing conditions depending on whether or not an adhesion layer exists in a 1 μm thin film.

**Figure 10:**
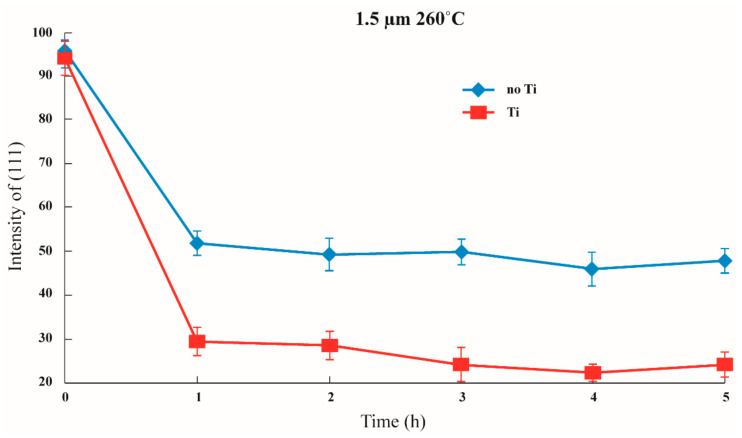
Changes of (111) intensity under the same annealing conditions depending on whether or not an adhesion layer exists in a 1.5 μm thin film.

Furthermore, as mentioned in the literature, when the thickness is lesser, the transformation to (100) is comparatively small. On the other hand, when the thickness is greater, it is easier to transform to (100). In addition, it was observed that the existence of the adhesion layer will increase the transformation ratio from (111) to (100). As shown in Figure 8, Figure 9 and Figure 10, when the thickness of the thin film was 0.5 μm, about 95% of the preferred orientation was at (111). On the other hand, when the thickness of the thin film was 1.0 μm, the preferred orientation at (111) decreased from approximately 75% to about 55%. Lastly, when the thickness was at 1.5 μm, it decreased from approximately 50% to about 25%, transforming from (111) to (100).

Although the figures cannot prove whether the existence of an adhesion layer will influence the energy, it has been proven that an adhesion layer can reduce the interfacial energy [34]. The 95% texture orientation of (111) at the beginning shows that the adhesive capacity significantly increased. Therefore, it can be concluded that the critical thickness for texture transformation could occur with or without an adhesion layer. Moreover, the transformation intensity was enhanced with the existence of a Ti adhesion layer and the increase of the thickness. At the same time, when the thickness of the silver film overreaches the critical thickness, the texture transformation of the thin Ag film has a considerable change, but the long annealing time does not determine the texture transformation.

### 3.3. Influences of an Imposed Initial Stress

**Figure 11:**
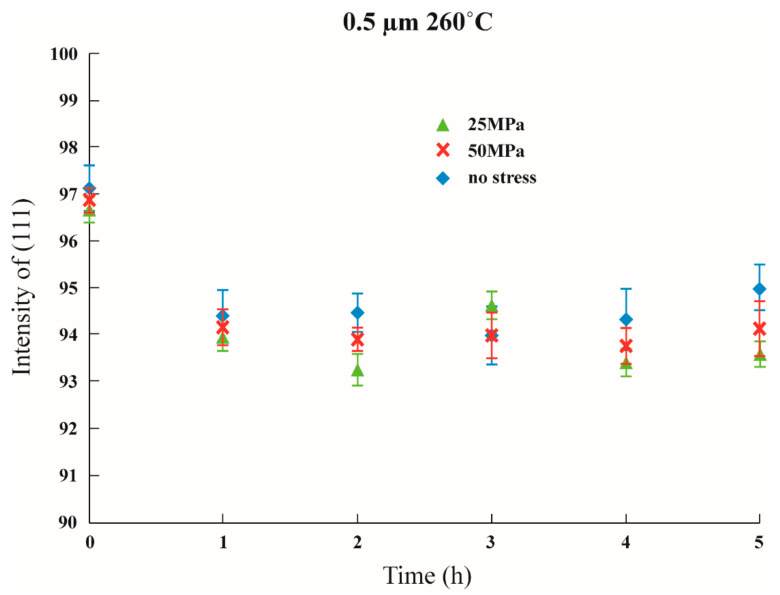
The intensity of (111) under distinctive stresses and after annealing 260 °C with a 0.5 μm thin film without Ti.

**Figure 12:**
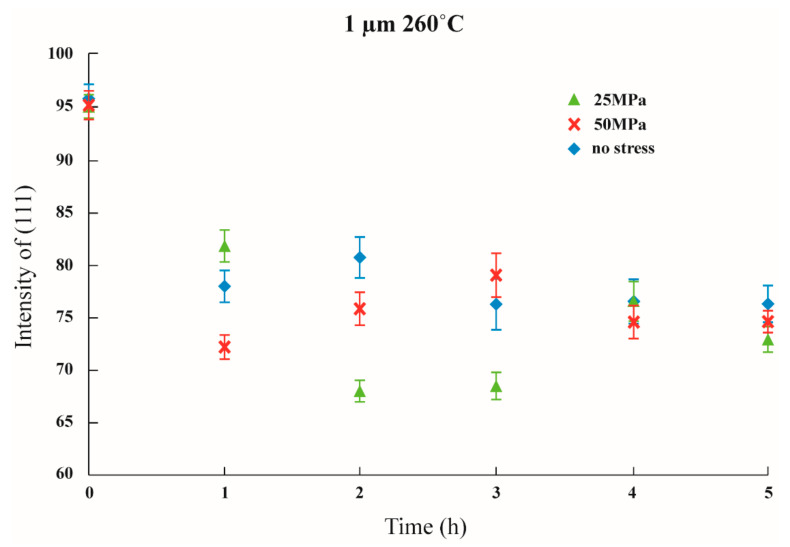
The intensity of (111) under distinctive stresses and after annealing at 260 °C with a 1 μm thin film without Ti.

**Figure 13:**
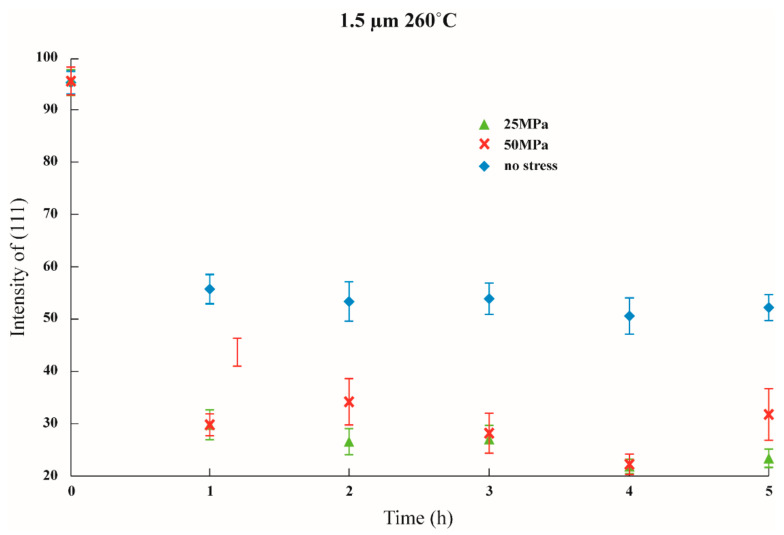
The intensity of (111) under distinctive stresses and after annealing at 260 °C with a 1.5 μm thin film without Ti.

**Figure 14:**
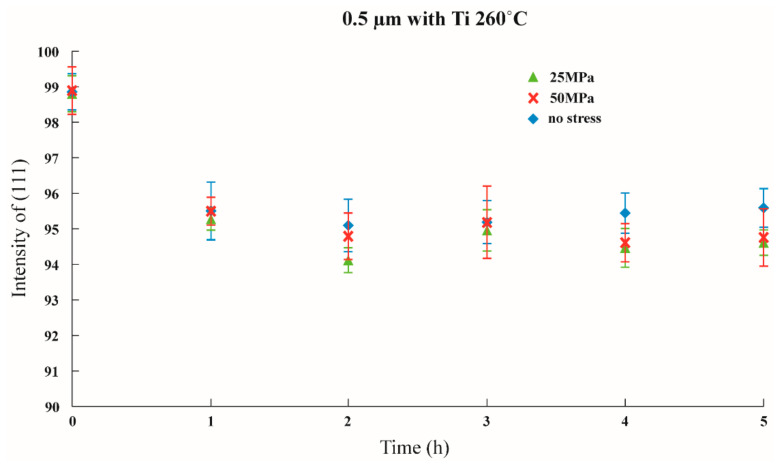
The intensity of (111) under distinctive Stresses and after annealing at 260 °C with a 0.5 μm thin film with an adhesion layer.

**Figure 15:**
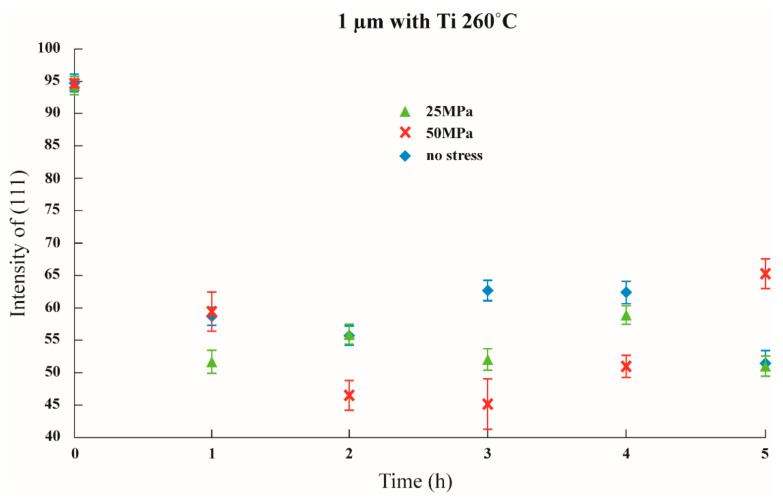
The intensity of (111) under distinctive stresses and after annealing at 260 °C with a 1 μm thin film with an adhesion layer.

**Figure 16:**
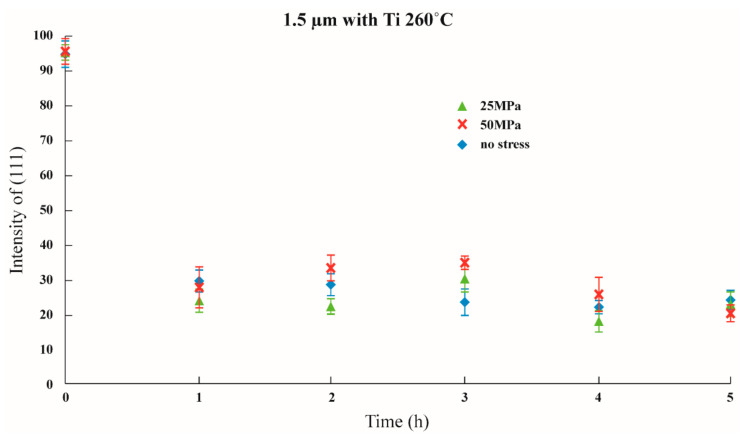
The intensity of (111) under distinctive stresses and after annealing at 260 °C with a 1.5 μm thin film with an adhesion layer.

Initial stress was imposed to observe whether the texture transformation can be influenced when the film is subjected to initial stress. The results are shown in Figure 11, Figure 12 and Figure 13 in which the samples did not have an adhesion layer, and in Figure 14, Figure 15 and Figure 16, in which an adhesion layer was added. When the thickness of the films was at 0.5 μm, with no adhesion layer, the intensities of (111) were all at 95%. When an adhesion layer existed, the proportion was comparatively high when the deposition had recently been completed, and the intensity remained at 95% after annealing.

Therefore, when the thickness of the films was 0.5 μm, all the main texture structures were at (111). When the thickness increased to 1 μm, with no adhesion layer, the intensity of (111) varied from 70% to 80%, and when an adhesion layer existed, after annealing, all intensities were between 50% and 60%. The imposition of initial stress had no significant influence on 1 μm films. Finally, the thickness was increased to 1.5 μm; with no adhesion layer, the intensity of (111) was between 30% and 50%. Although the texture transformation speed was faster under the initial stress, all the crystal orientations were transformed to (100).

When an adhesion layer existed, regardless of the existence of the initial stress, the intensity of (111) saw no significant changes, which varied from 25% to 35%. Therefore, when the thickness of the films was 1.5 μm, all the texture orientations were at (100). These results demonstrated the unique phenomenon that, when the thickness of the film is 1.5 μm, whether an adhesion layer is added or not or whether initial stress is imposed, and with an intensity of about 30%, all texture orientations will reach (100).

When the thickness of the film was less than the critical thickness, the imposition of initial stress had no significant influence on the transformation of the texture, and the main texture was the same as that under the same thickness. Similar results were postulated by a study that provided the initial stress on thin Ag film using bulge experiments [8].

### 3.4. Internal Stress in Metal Thin Film during Annealing

During annealing, internal stress is inevitable since the thin films experience temperature changes, and the substrates and film have distinctive coefficients of thermal expansion. The coefficient of the thermal expansion of silicon is 3 × 10^−6^ °C [31], while the coefficient of the thermal expansion of Ag is 18.9 × 10^−6^ °C. The initial temperature was set at 25 °C, with the experimental temperature set at 260 °C. After calculation using Equation (4), the strain value caused by the distinctive thermal expansion coefficients was 0.0037365. The stress values of (111) and (100) were calculated using Equation (5).

(4) $$\varepsilon_{misfit}=\left(\alpha_{film}-\alpha_{substrate}\right)\left(T_{f}-T_{s}\right)$$

(5) $$\sigma =\frac{Ε}{1-\nu }\varepsilon$$

where

ν stands for Posson’s ratio equal to 0.37,

Ε stands for Young’s modulus equal to 174 GPa at (111) and 76 GPa at (100) [35], and

ε is substituted by the value obtained in Equation (4).

Finally, we calculated that $\sigma^{111}$ is equal to 1.032 MPa, and $\sigma^{100}$ is equal to 450.75 MPa. The thin films may also be influenced by the strain caused due to the distinctive in the grain size before or after annealing, resulting in changes of texture orientation. This was calculated using Equation (6).

(6) $$\varepsilon_{misfit}=\delta \left(\frac{1}{d_{i}}-\frac{1}{d_{f}}\right)$$

where

$d_{i}$ is the initial grain size number, and

$d_{f}$ is the final grain size number.

The grain size number can be calculated by measuring the amount of growth the grain sizes had after annealing as described in the following.

### 3.5. Measurement Results from EBSD

**Figure 17:**
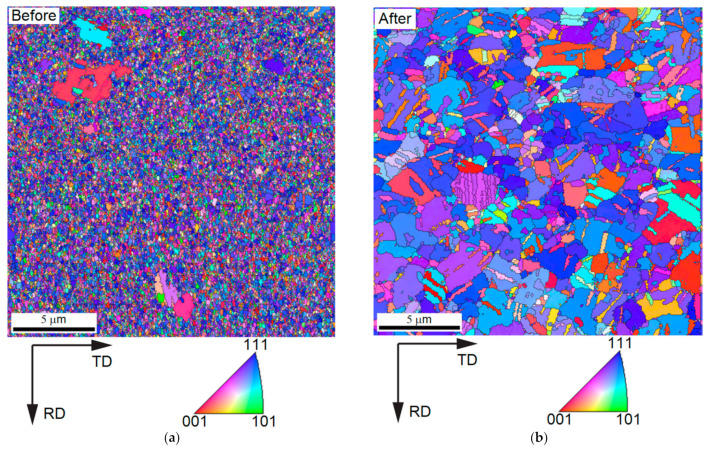
Inverse Pole Figure of 1.5 μm Thin Ag Film before Annealing Measured by EBSD (**a**) Before and (**b**) After.

**Figure 18:**
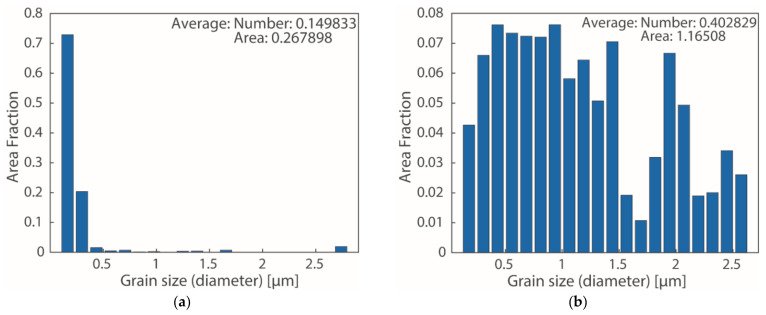
Grain Size Distribution Graph of 1.5 μm Thin Ag Film Annealing Measured by EBSD (**a**) Before and (**b**) After.

**Figure 19:**
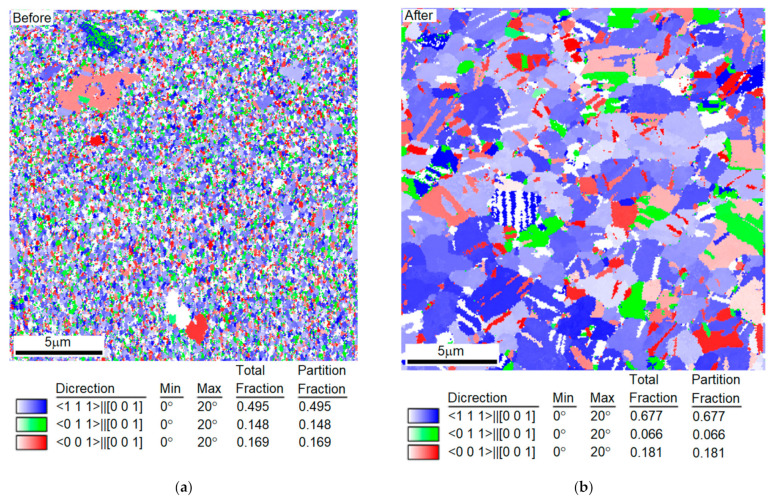
Distribution Graph of Each Crystallographic Direction of 1.5 μm Thin Ag Film after Annealing Measured by EBSD (**a**) Before and (**b**) After.

EBSD was used to observe the grain size changes after annealing. Figure 17, Figure 18 and Figure 19 compare the grain sizes of the 1.5 μm Ag thin films before and after annealing for one hour at 260 °C. We discovered that, due to recrystallization after annealing, the grain sizes significantly increased. As shown in Figure 18, the grain size was 164.28 nm before annealing, which increased to 433.35 nm after annealing. Consequently, the strain value produced by the growth of grains was 5.4802 × 10^−4^, which was calculated using Equation (6). By substituting this value into Equation (5), the values of $\sigma^{111}$ and $\sigma^{100}$ obtained were 95.36 and 41.65 MPa, respectively.

For the test specimens that experienced a temperature increase from 25 to 260 °C through heat treatment, the stress $\sigma^{111}$ increased to 151.36 MPa as the grains grew during the temperature increase. Consequently, the total stress was 1.18 GPa. When the test specimen temperature returned to 25 °C, the stress $\sigma^{100}$ was 450.75 MPa, and the stress $\sigma^{100}$ after grain growth was 66.11 MPa.

After calculation, the total stress was 666.5 MPa. Compared to the 25 and 50 MPa given by the experiment, the value of the internal stress was far greater. Consequently, the result was consistent with Section 3.3, i.e., during the annealing process, the initial stresses imposed on the films in the experiments here were small and did not reach the threshold to significantly influence the transformation of the texture structure.

### 3.6. Discussion

The research on the texture transformation of thin Ag films was under two conditions: (1) with or without an adhesion layer in the thin film and (2) with or without initial stress applied through a four-point bending load. In the experiment, two samples (silicon/silver and silicon/titanium/silver) were used to apply different initial stress/strain values and different annealing times.

Previously, with the Carl, Thompson, and Frost (CTF) model [27,28,29], the transformation from (111) to (100) was based on the competition between interfacial energy and strain energy. The orientation of (111) has the lowest interfacial energy, while (100) has the smallest biaxial modulus and the smallest strain energy. When a fixed elastic strain is given, changes in the thickness of the thin film indicate the reduction of the interfacial energy for each unit volume, with the strain energy per unit volume and the thickness of the film being constant. Consequently, there exists a critical thickness; if the film is thicker than the critical thickness, regardless of the influence from grain size, the initially preferred orientation of the thin film (111) will be transformed to (100) completely.

In this study, transitions from (111) texture at low thicknesses to (100) at high thicknesses were indeed observed as shown in Figure 5 and Figure 6. However, the transition is typically observed to occur gradually over a range of thickness, with films having both (111) and (100)-oriented grains (mixed texture) in the transition region of film thickness.

It could argue that mixed texture is the result of a kinetically limited intermediate state and that the transformation would proceed to its equilibrium endpoint if the films were annealed for longer or at higher temperatures. However, our experimental results (Figure 8, Figure 9 and Figure 10) suggested that this type of mixed texture is quite stable and will not be transformed completely. As a result, the existing thermodynamic model is deficient in some respects when it comes to predicting texture.

In order to resolve these deficiencies, we closely investigated the behavior of the transformation mechanism in the presence of the adhesion layer. The test samples were annealed at the most suitable temperature of 260 °C according to previous studies [24]. We observed that the existence of the adhesion layer increased the transformation ratio from (111) to (100). As shown in Figure 8, Figure 9 and Figure 10, when the thickness of the thin film was 0.5 μm, about 95% of the preferred orientation was at (111). On the other hand, when the thickness of the thin film was 1.0 μm, the preferred orientation at (111) decreased from approximately 75% to about 55%.

Lastly, when the thickness was at 1.5 μm, it decreased from approximately 50% to about 25%, transforming from (111) to (100). It appears that the presence of a Ti adhesion layer increased the transformation amount of (111) to (100) texture. Similarly, Arzt et al. [22,29,30,31] discovered that, for film deposits on different substrates, the transformation mechanism was distinctive from the CTF model. In particular, films with a Ti adhesion layer showed a sharper texture transition. We speculate that the relatively lower surface energy and soft lattice constrain on Ti than Si could be the reason for the sharper texture transition.

Vodnick et al. [36] suggested that the insufficiency in the CTF model was because it fails to account for the inhomogeneous three-dimensional stress states that develop in films with mixed texture. The stress states affect both the thermodynamics of the problem by determining which grain orientations are stable locally in a film and also determine the kinetics of texture transformations by changing the driving force mechanism dynamically during microstructure evolution.

Since it is true that a film has mixed texture, complex three-dimensional stress states will occur due to the requirement of stress field continuity [36]. If a uniform equal biaxial elastic strain could be imposed in the plane of the film, the stresses in the texture components would scale with the biaxial moduli in each, leading to large stress discontinuities at the texture boundaries. This discontinuity can be eliminated by elastic bowing of the texture boundaries by interface sliding near the texture boundary or by differential plastic deformation in the adjacent texture components.

In all cases, these accommodations need be made only near texture boundaries; therefore, one expects the in-plane stresses in the texture components to depend strongly on the aspect ratio of the texture regions. If the spacing between texture boundaries is much larger than the film thickness, the in-plane stresses can be quite different in the different texture components depending on the elastic and plastic properties. On the other hand, if the texture boundary spacing is comparable to or smaller than the film thickness, then accommodations near texture boundaries must lead to in-plane stresses that are the same in the texture components.

We studied the initial stress effect of 25 and 50 MPa on samples under four-point bending experiments. From these results, the transformation of 0.5 µm thickness of thin Ag films with and without an adhesion layer did not occur or occurred only to a minimal extent when stress was applied. However, when the thickness of the film was 1.5 μm, whether an adhesion layer was added or not or whether initial stress was imposed, and with an intensity of about 30%, all the texture orientation reached (100). As indicated in 3.5, for the test specimens that experienced a temperature increase from 25 to 260 °C, the total stress was 1.18 GPa.

When the test specimen temperature returned to 25 °C, the total stress was 666.5 MPa. Compared to 25 and 50 MPa given by the experiment, the value of the internal stress was far larger. A study by Ellis, E. A. et al. [8] suggested that the influence of stress is distinctive from the driving force description in the CTF model. They indicated the initial stress in the range of 100 MPa could be too small compared to the internal stress; thus, the initial stress imposed here was not significantly influenced by the texture transformation.

It should also be noted that the texture transformation of the thin metal film after EBE deposition must be maintained at a temperature lower than room temperature through the study, which is required to hold the texture of the thin metal film unchanged. In summary, neither the stress nor the interface energy plays a dominant role in the texture transformation of Ag. We investigated the driving forces involved in this transformation by using a four-point bend testing apparatus to induce different stresses in thin Ag films under identical annealing conditions with different substrates. The results all indicated that the transformation mechanism of Ag films cannot be simplified as with the conventional CTF model.

In summary, we posit that, in thin Ag films with normally or log-normally distributed grain sizes, some grains will be at an aspect ratio such that (100) is stabilized, while others with different aspect ratios will be stabilized in the (111) orientation. Thus, it may be true that the CTF thermodynamic equilibrium would predict a single texture component. However, the mixed texture occurs not because of kinetic limitations due to grain boundary grooving or solute drag, but because the driving force is locally reduced due to the interaction of the grain size, grain orientation, and the complex three-dimensional stress state. Further research on surface of the films by AFM and SEM and cross-section SEM incorporated with XRD should be performed in the future.

## 4. Conclusions

In this study, we discussed the influence of imposed initial stresses and different interfaces on the texture transformation of thin metal films. Whilst ensuring that the distinctive conditions of the imposed initial stresses and annealing times were under control, this study carried out annealing at 260 °C on two types of test specimens (Si/Ag and Si/Ti/Ag) to observe the level of influence that different amounts of initial stress and the existence of an adhesion layer had on the transformation mechanism of the texture orientation of thin Ag films. We discovered that the deposition methods for thin films influenced their initial texture.

Since the texture structure of thin films was related to the substrate temperatures and the pressures of a working environment, we set up a consistent working environment to observe the differences in the texture transformation of thin films after annealing. Subsequently, this study explored the influence of the existence of a Ti adhesion layer. It was observed that the transformation ratio increases as the thickness increases. Regardless of the existence of the adhesion layer, texture transformation occurred, and, for thin films with a thickness of 1 and 1.5 μm and Ti adhesion layers, the texture transformation was relatively significant.

On the other hand, concerning the imposition of initial stress, we found that the amount of the stress did not influence the texture transformation. The reason for this could be that the initial stress imposed in the study was too small compared to the internal stress. According to the CTF thermal model, the main factors that drive the texture changes of a thin film lie in the strain energy and surface/interfacial energy. The experimental results showed that the CTF model was not sufficiently accurate to explain the texture transformation demonstrated in this study. There exist certain other factors, such as the driving force due to the recrystallization process to replace existing defects of dislocations, vacancies, and twin boundaries [3,24,36], that could lead to texture transformation in thin films after annealing.

## Figures and Tables

**Table 1:** Parameters of depositing thin silver film using an E-gun.

Background Pressure	Deposition Rate	Temperature of the Deposit Substrate	Target Material
3~5 × 10^−7^ Torr	25 Å/s	35~60 °C	99.99% Ag

**Table 2:** Parameters of depositing thin titanium film by sputter.

Background Pressure	Working Pressure	Ar Gas Flow	Power	Deposition Rate	Target Material
3~5 × 10^−7^ Torr	10^−3^ × 10^−4^ Torr	15 sccm	250 W	5.5 Å/sec	99.99% Ti

**Table 3:** Parameters of the experimental test specimens.

Material	Young’s Modulus	Initial Stress Imposed	Test Specimen Size
Silicon Wafer (400 μm)	130 Gpa	44 Gpa	b = 5.0 mm
Thin Silver Film	76 Gpa	25 Gpa	a = 6.5 mm

## Data Availability

Not applicable.
